# Investigation of the cleaning performance of commercial orthodontic cleaning tablets regarding biofilm removal on PMMA test specimens: An ex vivo study

**DOI:** 10.1007/s00056-023-00474-2

**Published:** 2023-06-02

**Authors:** A. Arndt-Fink, P.-G. Jost-Brinkmann

**Affiliations:** CharitéCenter 3 for Oral Health Sciences (CC 3), Department of Orthodontics and Dentofacial Orthopedics, Charité—Universitätsmedizin Berlin, Corporate Member of Freie Universität Berlin and Humboldt-Universität zu Berlin, Aßmannshauser Str. 4–6, 14197 Berlin, Germany

**Keywords:** Polymethyl methacrylate, Removable orthodontic appliances, Removal of biofilms, Denture hygiene, o‑phthaldialdehyde (OPA) method, Polymethylmethacrylat, Herausnehmbare kieferorthopädische Apparaturen, Biofilmentfernung, Prothesenhygiene, OPA(o-Phthaldialdehyd)-Methode

## Abstract

**Purpose:**

The purpose of this ex vivo study was to compare the cleaning performance of three commercially available orthodontic cleaners on polymethyl methacrylate (PMMA) test specimens covered with biofilm.

**Methods:**

Twenty subjects wore an individually manufactured vacuum-formed maxillary splint with four integrated PMMA test specimens for 7 days. The four test specimens were located on the buccal surfaces of the maxillary molars. After a 7-day wearing period, the PMMA test specimens colonized by biofilm were divided into two halves. One half was placed in 150 ml of tap water or in 150 ml of cleaning solution of the cleaners Retainer Brite® (Dentsply International Raintree Essix, Sarasota, FL, USA), Kukis® Xpress (Reckitt Benckiser, Heidelberg, Germany) or Dontodent (Propack, Heidelberg, Germany) while the other half remained uncleaned. The modified o‑phthaldialdehyde (OPA) method was used to determine the amount of protein on both halves of the test specimens. The difference was tested for significance as a measure of the cleaning effect using a paired sample t‑test.

**Results:**

The cleaning performance of the three orthodontic cleaners was higher than the cleaning performance of tap water (mean 25.9 ± 6.5%). While Retainer Brite® (mean 54.5 ± 7.1%) removed significantly more biofilm than Dontodent (mean 41.5 ± 9.2%, *p* < 0.001) and Kukis® Xpress (mean 39.9 ± 11.5%, *p* < 0.001), there was no significant difference in the cleaning performance between Kukis® Xpress and Dontodent (*p* = 1).

**Conclusion:**

Seven-day-old biofilm is only removed partially by the investigated orthodontic cleaners, so that they are not suitable as the only measure for removing established biofilms.

## Abstract (de)

**Ziel:**

Ziel dieser Ex-vivo-Studie war ein Vergleich der Reinigungswirkung von drei kommerziell angebotenen Zahnspangenreinigern auf Biofilm-behaftetem PMMA(Polymethylmethacrylat)-Kunststoff.

**Material und Methode:**

20 Proband*innen trugen 7 Tage lang eine Schiene mit 4 integrierten PMMA-Prüfkörpern bukkal im Bereich der oberen Molaren. Nach 7 Tagen Tragezeit wurden die von Biofilm besiedelten PMMA-Prüfkörper halbiert. Eine Hälfte wurde in 150 ml Leitungswasser oder in 150 ml Reinigungslösung der Reiniger Retainer Brite® (Dentsply International Raintree Essix, Sarasota, FL, USA), Kukis® Xpress (Reckitt Benckiser, Heidelberg, Germany) oder Dontodent (Propack, Heidelberg, Germany) gelegt, während die andere Hälfte ungereinigt blieb. Mit der modifizierten OPA(o-Phthalaldehyd)-Methode wurde die Proteinmenge auf beiden Prüfkörperhälften bestimmt. Die Differenz wurde als Maß der Reinigungswirkung mittels t‑Test für verbundene Stichproben verglichen.

**Ergebnisse:**

Die Reinigungsleistung der drei Spangenreiniger war höher als die Reinigungsleistung von Leitungswasser (Mittelwert 25,9 ± 6,5 %). Während Retainer Brite® (Mittelwert 54,5 ± 7,1 %) signifikant mehr Biofilm entfernte als die beiden Reiniger Dontodent (Mittelwert 41,5 ± 9.2 %, *p* < 0,001) und Kukis® Xpress (Mittelwert 39,9 ± 11,5 %, *p* < 0,001) bestand kein signifikanter Unterschied in der Reinigungsleistung zwischen Kukis® Xpress und Dontodent (*p* = 1).

**Schlussfolgerung:**

Sieben Tage alter Biofilm wird von den untersuchten Zahnspangenreiniger nur unzureichend entfernt, sodass diese als alleiniges Mittel zum Entfernen etablierter Biofilme nicht ausreichen.

## Introduction

Removable orthodontic appliances (ROAs) are used for active treatment and retention. ROAs consist of wire elements and mostly of polymethyl methacrylate (PMMA) [14, 19]. Within minutes of inserting an appliance into the oral cavity, the surfaces of the appliance become colonised with bacteria [33]. They associate together to form biofilms, a complex matrix-like structure and harbour pathogens such as bacteria, viruses, fungi and protozoa [23]. Bacteria of the oral flora are the cause of local diseases such as periodontitis, peri-implantitis and dental caries [5, 17, 29]. The risk of caries can increase during the use of ROAs as they can promote for the colonisation of *Streptococcus mutans* [6, 38] and lactobacilli [40, 42] in the oral cavity. Furthermore, oral bacteria can be the cause of systemic diseases and are associated with neurodegenerative diseases such as Alzheimer’s disease [11, 26, 37, 39]. Consequently, removing biofilm from these appliances and removable partial dentures is of great importance.

Common cleaning methods include manual cleaning with a toothbrush, often in combination with toothpaste or dishwashing liquid, as well as chemical cleaning with cleaning tablets and immersion in disinfectant solutions or acetic acid [12].

According to Diedrich [10] especially in hard-to-reach areas such as around wire elements embedded in acrylic and screws, mechanical cleaning is insufficient, while cleaning tablets lead to better cleaning.

Based on their active ingredients, chemical denture cleaners can be classified into alkaline peroxides, alkaline hypochlorites, acids, disinfecting agents and enzymes [7, 32]. Alkaline peroxides are often available in form of tablets. They produce an effervescent alkaline solution that generates hydrogen peroxide and active oxygen when added to water. This process leads to disinfection and oxidation of coloured deposits. In the presence of acids, sodium bicarbonate reacts forming carbon dioxide and creating a foam. Furthermore, the rising gas bubbles create a mechanical component. Substances such as surfactants serve as an additional cleaning component, and the addition of potassium monopersulfate has bactericidal, fungicidal and virucidal effects [7, 34]. Other ingredients include fillers as well as colouring and flavouring agents, which are intended to give the consumer a feeling of freshness.

The use of cleaning tablets in addition to mechanical cleaning is recommended to their patients by 70% of Greek [41] and 72% of Turkish orthodontists [2], while 37% of German orthodontists recommend cleaning with chemical adjuvants only [13]. In previous studies on the performance of orthodontic cleaners, cleaning efficiency was investigated on soft plaque which accumulated within a few hours or days [3, 9, 16, 30]. Yet, it has remained unclear whether orthodontic cleaners are effective on a 7-day-old established biofilm and its associated structural changes. The aim of the present study was to clarify this question.

## Materials and methods

### Sample size calculation

Sample size calculation was done with the procedure MOE1‑1 of nQuery 8.6.1.0 prior to recruitment based on an earlier pilot study [16]. As three cleaners were tested, the multiple one-sided significance level was adjusted from alpha = 0.025 to alpha = 0.0071. It was further assumed that the cleaning performance of all three products is at least 80% and that the dispersion of the cleaning performance amounts to sigma = 6.1%. The one-sided one-sample t‑test against mu0 = 75% has a power of at least 80% with a sample size of *n* = 20.

### Study cohort

All participants of this study were informed in advance about the purpose of the study and possible risks in both verbal and written form. They gave their written consent to participate in the study. A total of 20 healthy, adult participants (11 women and 9 men, mean 34.5 years, range 19–61 years) were recruited.

All participants included in this study agreed to wear a test device in the form of a vacuum-formed splint for 7 days. The splint was also worn while eating and was only allowed to be removed for toothbrushing with a non-fluoride toothpaste. The splint was not allowed to be cleaned; only food remnants were allowed to be removed carefully without touching the surfaces of the PMMA test specimens. The participants consented to not using fluoride-containing preparations or antibacterial mouth rinses containing chlorhexidine or essential oils during the 7‑day study period. Exclusion criteria for participation were severe general diseases, active caries or periodontitis, the absence of first or second molars in the upper jaw or allergies to PMMA/MMA. The use of oral antibiotics during the wearing period or within the last 3 months also led to exclusion from the study.

### Test device

**Fig. 1:**
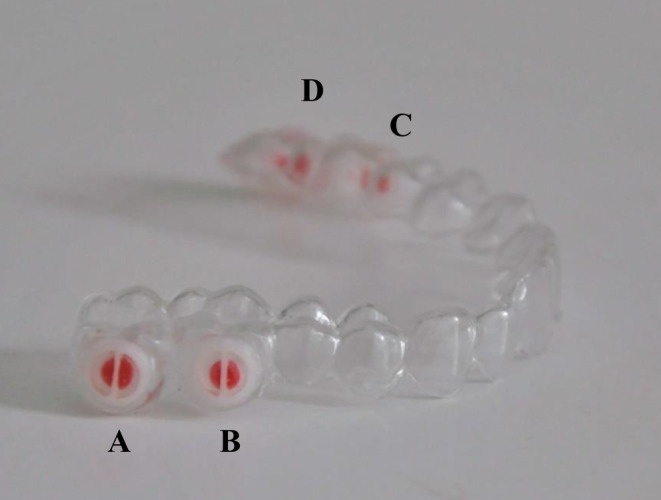
Vacuum-formed maxillary splint with four integrated test specimens located on the buccal surface of the molars. The access to the test specimens was covered with wax for better visualisation and replaced with silicone for the trials. The location of the test specimens was numbered with the letters *A*–*D*. The allocation of the cleaners to the test specimens rotated according to a predefined protocol Tiefziehschiene für den Oberkiefer mit 4 Prüfkörpern lokalisiert an den bukkalen Flächen der Molaren. Der Zugang zu den Prüfkörpern wurde zur besseren Visualisierung mit Wachs abgedeckt und für die Probandenversuche durch Silikon ersetzt. Die Lokalisation der Prüfkörper wurde mit den Buchstaben *A*–*D* nummeriert. Die Zuordnung der Reiniger zum Prüfkörper rotierte nach einem vorher festgelegten Protokoll

The test device contained four identical PMMA test specimens in an individual manufactured vacuum-formed splint for the upper jaw. For this purpose, an alginate impression was taken of the upper jaw and models were made of hard dental stone. A 1 mm, hard-elastic and transparent splint (DURAN®, Scheu-Dental, Iserlohn, Germany) was pressure-moulded over the plaster models of the study participants. The PMMA test specimens were produced separately. For this purpose, the holders for the test specimens were printed using a standardised three-dimensional (3D) printing process from polylactic acid (PLA; 7 mm, 5 mm, 1.5 mm). The inner surface of each PLA holder was designed to be conical (7°) and thus prevented the test specimen halves from falling out of the holder. The PLA ring was split in the middle by a thin partition wall, creating two identically dimensioned test specimen halves. The PLA holders were coated with a separating agent (3D Isoliermittel, Dentaurum, Germany) and then filled with PMMA (Orthocryl®, Dentaurum, Ispringen, Germany). To achieve an even surface, the test specimens were placed between two glass plates, loaded by a constant weight (500 g) and then polymerised in a pressure pot at 2.2 bar and 40 °C. The test specimens were then placed symmetrically on the buccal surfaces of the maxillary molars and firmly bonded to the splint using Orthocryl LC® (Dentaurum, Ispringen, Germany; Fig. 1). To achieve a stable bond, the surrounding areas of the splint were first roughened with sandpaper and covered with monomer. Four holes, previously drilled in the middle of the molar range (diameter 3 mm) served as an access to the two halves of the test specimens. During the wearing period, the holes in the DURAN® splint were covered with silicone so that only the buccal surfaces of the test specimens were colonised by biofilm. The device was finished like a retention splint and then inserted. At the end of the 7‑day wearing period, the contaminated splints were collected and were promptly examined using the modified o‑phthaldialdehyde (OPA) method.

### Cleaning of the test specimens

Four test specimen halves of every participant were each placed in tap water (control medium) and respectively in the cleaning solution of the following three cleaning tablets: Retainer Brite® (Dentsply International Raintree Essix, Sarasota, FL, USA), Kukis® Xpress (Reckitt Benckiser, Heidelberg, Germany) and Dontodent (Propack, Heidelberg, Germany). The cleaning tablets were each added to a glass filled with 150 ml water at a temperature of 40 ± 2 °C. The PMMA test specimen halves to be cleaned were simultaneously placed in the bubbling cleaning solution. The immersion time of the PMMA test specimens in the cleaning solution was, according to the manufacturers’ time instructions, 3 min for Kukis® Xpress, 10 min for Dontodent and 15 min for Retainer Brite® and tap water. The uncleaned half of each test specimen was used to determine the amount of protein before cleaning. After completing the cleaning time, the cleaned test specimen halves were transferred to a sample tube filled with 500 µl of 1% sodium dodecyl sulfate (SDS) solution and shaken for 30 min in an ultrasonic bath at 30 °C. The protein solution was transferred to a cuvette and the extinction was measured photometrically. Afterwards, 500 µl of OPA reagent were added to the solution. The OPA reagent was prepared daily and had to be replaced by a newly prepared solution as soon as the absorbance was not in the range of E = 0.641 ± 0.032, measured against a leucin standard solution. For this purpose, 0.04 g o-phthaldialdehyde and 1 ml methanol were stirred in an Erlenmeyer flask with the aid of a magnetic stirrer. To the homogeneous solution 0.116 g 2‑mercaptoethanesulfonic acid was added (solution A). In another Erlenmeyer flask, 50 ml aqua dem. and 1.005 g disodium tetraborate were stirred with the aid of a magnetic stirrer (solution B). This was followed by the transfer of solution A into solution B and the addition of 1.25 ml of 20% SDS solution.

### Protein quantification using the modified OPA method

Protein quantification as an indicator of contamination was determined for the cleaned and the uncleaned test specimen halves using the modified OPA method.

Free α‑ and ε‑terminal amino groups in amino acids, peptides and proteins react in the presence of a thiol component to form a fluorescent end product (1-alkylthio-2-alkylisoindoles). This product is spectrophotometrically detectable at 340 nm and quantifiable by measuring the extinction [21].

To quantify the amount of protein on the test specimens, calibration with bovine serum albumin (BSA, fraction V, Sigma Aldrich®, St. Louis, MO, USA) of known concentration was carried out previously. Several dilution standards were made from a prepared BSA solution (concentration 1000 µg/ml) and measured photometrically. A regression line, y = mx + b, was created using the measured absorbance values from a total of six measurement series. The quantification of the initially unknown concentrations of the protein samples was carried out using this regression line. The amount of protein detectable after cleaning compared to the detectable amount of protein of the uncleaned control group served as an indicator for the cleaning performance.

### Statistical analysis

Statistical Package for Social Sciences (SPSS version 27.0; IBM, Armonk, NY, USA) was used for all statistical analyses and graphical presentations. For the comparison of the cleaning performance of the three cleaners, a paired sample t‑test was used, with a *p*-value of ≤ 0.05 defined as significant. All *p*-values were adjusted by a factor of 3 after Bonferroni.

## Results

**Fig. 2:**
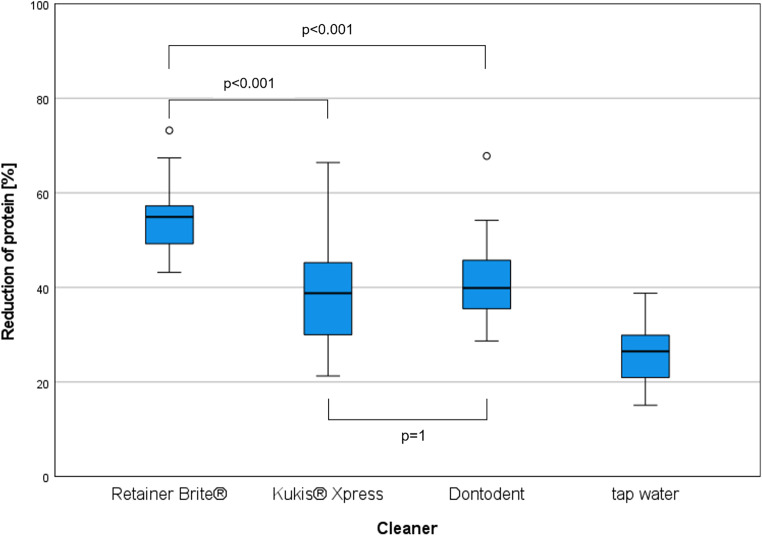
Boxplot diagram showing the reduction of protein in % on the surface of the polymethyl methacrylate (PMMA) test specimens after cleaning compared to the uncleaned control group. Mean values ± standard deviation: Retainer Brite® 54.5 ± 7.1%, Kukis® Xpress 39.9 ± 11.5%, Dontodent 41.5 ± 9.2% and tap water 25.9 ± 6.5%. Median values: Retainer Brite® 54.9%, Kukis® Xpress 38.8%, Dontodent 39.9% and tap water 26.5% Boxplot-Diagramm, das die Proteinreduktion in % auf der Oberfläche der PMMA(Polymethylmethacrylat)-Prüfkörper nach Reinigung im Vergleich zur ungereinigten Kontrollgruppe darstellt. Mittelwerte ± Standardabweichung: Retainer Brite® 54,5 ± 7,1%, Kukis® Xpress 39,9 ± 11,5%, Dontodent 41,5 ± 9,2% und Leitungswasser 25,9 ± 6,5%. Medianwerte: Retainer Brite® 54,9%, Kukis® Xpress 38,8%, Dontodent 39,9% und Leitungswasser 26,5%

The protein reduction of the three orthodontic cleaners and the control medium water is shown in a boxplot diagram (Fig. 2). The cleaning performance of the orthodontic cleaner Retainer Brite® (mean 54.5 ± 7.1%) was significantly higher than that of Kukis® Xpress (mean 39.9 ± 11.5%, *p* < 0.001) and Dontodent (mean 41.5 ± 9.2%, *p* < 0.001). There was no statistical difference in cleaning performance between the cleaners Kukis® Xpress and Dontodent (*p* = 1). The cleaning performance of the control medium water was 25.9% (Table 1).

**Table 1:** Descriptive statistics regarding cleaning efficacy and *p*-values Deskriptive Statistik der Reinigungsleistung und *p*-Werte

	Retainer Brite®	Kukis® Xpress	Dontodent	Tap water
*N*^a^	19	19	20	20
Mean ± SD	54.5 ± 7.1	39.9 ± 11.5	41.5 ± 9.2	25.9 ± 6.5
95% CI for mean	51.1; 57.9	34.3; 45.4	37.1; 45.8	22.8; 28.9
Minimum	43.2	21.3	28.7	15.1
Maximum	73.2	66.4	67.8	38.8
Median	54.9	38.8	39.9	26.5
Bonferroni *p* and (unadjusted *p*)	Retainer Brite®–Kukis® Xpress		*p* < 0.001*	(*p* < 0.001)
	Retainer Brite®–Dontodent		*p* < 0.001*	(*p* < 0.001)
	Kukis® Xpress–Dontodent		*p* = 1^b^	(*p* = 0.408)

*95% CI* 95% confidence interval, *SD* standard deviation

^a^Number of test specimens examined/investigated

^b^*p*-value adjusted by factor 3 according to Bonferroni, the result was a value of *p* > 1, which was then set to *p* = 1

*p < 0.05 indicates statistical significance

**Fig. 3:**
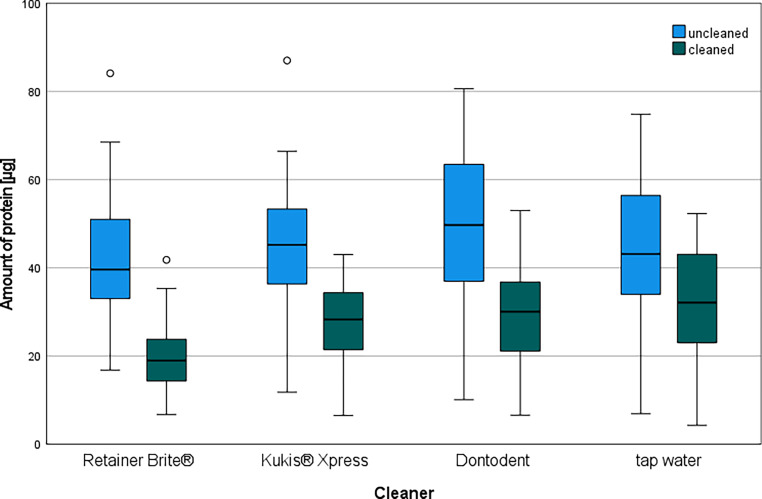
Boxplots illustrating the distribution of the amount of protein in the different cleaning groups before and after cleaning. values 1.5 × IQR (interquartile range) above the third quartile. *circles* Outliners Boxplots zur Darstellung der Verteilung der Proteinmengen in den verschiedenen Reinigergruppen vor und nach Reinigung. Werte 1,5 × IQR (Interquartilsabstand) über dem dritten Quartil. *Kreise* Ausreißer

The amount of protein of all uncleaned test specimens ranged from 6.9 to 87.0 µg; the protein amount of the cleaned test specimens ranged from 4.3 to 53.0 µg protein. The distribution of the protein amounts of all uncleaned and cleaned test specimens in relation to the cleaner used is shown in a boxplot diagram (Fig. 3). The median values of the protein amounts on the uncleaned test specimen halves ranged from 39.6 µg (Retainer Brite®) to 49.7 µg (Dontodent). The median values of the protein amounts on the cleaned test specimen halves ranged from 19.0 µg (Retainer Brite®) to 32.1 µg (tap water). The amount of protein on the uncleaned test specimen halves was higher than the protein amounts after cleaning using an orthodontic cleaning tablet or tap water. Tap water left the highest amounts of protein; Retainer Brite® left the lowest amount of protein.

## Discussion

Quantitative detection methods for proteins are, in addition to the OPA method, the BCA (bicinchoninic acid assay) method, the Bradford reagent and the Lowry method [25]. Furthermore, there are semiquantitative methods, such as the biuret reaction, and qualitative methods, such as the ninhydrin reaction [20]. The modified OPA method is a suitable method for the quantitative detection of proteins. It has a high sensitivity, with a detectability down to the picomole range [22], which enabled the use of small test samples in this study. However, very low absorbance values led to a limitation of the method in the present study. At values below 2 µg/ml BSA, the detection limit of the photometer (Shimadzu™ UVmini-1240, Kyoto, Japan) was approached, thus, especially the quantification of small protein amounts became increasingly inaccurate. This problem can be solved by generating higher extinction values that lie in the noncritical measurement range. For example, larger test specimen surfaces with resulting higher accumulation of biofilm could have been used. It is also possible to adjust the ratio of protein solution to OPA solution. However, the absorbance values of the samples in this study were not in the critical measurement range with concentrations above 8.7 µg/ml, so an adjustment was not necessary.

The OPA method was proven to be suitable in this study, and it can be concluded that quantitative protein measurement methods are appropriate for investigating the cleaning performance of orthodontic cleaners. Thus, protein measurement methods can be an alternative to common techniques such as analysis by scanning electron microscopy [3, 30].

The use of the test device, a thin vacuum-formed maxillary splint, that served as a holder for the test specimens was satisfactory. From previous investigations, it was found that the amount of protein on two closely spaced areas, in this case two halves of the test specimens, is almost identical. The assignment of the cleaners to the test specimens was rotated in order to compensate locally existing individual differences in the accumulation of biofilm between the participants. During the 7‑day wearing period, out of a total of 80 test specimens, only 2 were lost prematurely. For future studies, it is possible that the test device, consisting of the splint together with the holders, is 3D printed in a single piece.

Fixed orthodontic appliances facilitate plaque accumulation and complicate adequate daily plaque removal by toothbrushing [36]. During treatment with multibracket appliances especially patients with poor oral hygiene show a high prevalence of *Candida albicans*, *Streptococcus mutans* and lactobacilli [28]. From a caries-risk point of view ROAs are considered an alternative to fixed appliances as they can be removed by the patient and oral hygiene can be carried out more easily. The clinical consequences, including plaque and inflammation of the gingiva, are lower and there is a lower risk of the development of caries compared to treatment with fixed appliances [1, 31]. However, shortly after being inserted into the oral cavity, ROAs are colonised by biofilm harbouring pathogens [33]. ROAs colonised by biofilm, in turn, increase the risk of caries and gingivitis [4, 6, 35]. Consequently, it is important that any orthodontic appliance that is inserted into the oral cavity is clean and as free as possible of bacteria. The aim of the present ex vivo study was the recommendation of an optimal product that would eliminate an established biofilm and help to reduce the development of caries and the rates of gingivitis in patients with removable orthodontic appliances. However, the question which product sufficiently removes biofilm and how much biofilm a product may leave on an appliance and still may be considered an acceptable cleaning product remains open. The point at which cleaning can be described as sufficient has not yet been standardised internationally and there are no binding standards for the cleaning performance of chemical denture cleaners.

The literature shows that the cleaning performance of orthodontic cleaning tablets has already been the subject of previous studies [3, 9, 16, 30]. Other methods for cleaning orthodontic appliances, such as manual cleaning with a toothbrush or cleaning with antiseptic mouth rinses, have often been compared alongside chemical cleaning tablets. A study published in 2015 investigated the cleaning efficacy of three different cleaning methods on thermoplastic aligners. The combination of chemical cleaning tablets followed by manual cleaning was superior to both manual cleaning with toothpaste and manual cleaning without toothpaste [30]. Similar results were obtained in a recent study published in 2021, in which the combination of manual cleaning with chemical cleaning tablets led to the highest biofilm reduction [15].

Coimbra et al. [9] investigated the impact of peroxide-based solutions on multispecies biofilm formed by *Candida albicans, Staphylococcus aureus* and *Pseudomonas aeruginosa*. The biofilm was formed in vitro on acrylic resin specimens within 24 h. Cleaning tablets had an antimicrobial effect but did not promote widespread removal of the aggregated biofilm. The cleaning performance of chemical cleaning tablets, without the additional use of a mechanical component, was investigated in a pilot study by Fathi et al. [16]. They tested the cleaning performance of cleaning tablets on soft plaque that had accumulated in vivo over 4 days. The investigated orthodontic cleaners led to a protein reduction between 79.9% (Kukis®) and 86.8% (fittydent super®), while water led to a protein reduction of 56.5%. The question of whether an orthodontic appliance contaminated by a longer, undisturbed biofilm formation is equally effectively cleaned by orthodontic cleaners remained open and was the aim of this study.

The inferior ability of orthodontic cleaners to reduce protein levels in the present study compared to Fathi et al. [16] seems to be a consequence of the maturation of the biofilm and its associated structural changes [18]. Several mechanisms are responsible for the increasing resistance of mature biofilms to physical and chemical stresses. In addition to the proliferation of anaerobic bacteria, there is an overall increase in all bacteria and an increase in the heterogeneity of bacterial composition [27]. This enables the exchange of metabolic products, signalling molecules, genetic material and defence substances [18]. Extracellular polymeric substances (EPS) represent a diffusion barrier for antimicrobial substances by slowing down the transport speed into the interior of the biofilm or impairing it through interaction with extrapolymeric substances. This makes it increasingly difficult for antimicrobial substances to penetrate into deep layers of the biofilm [12, 24].

In this study, Retainer Brite® achieved the highest protein reduction and Kukis® Xpress and Dontodent achieved an equally significantly lower protein reduction. The cleaning performance of all three orthodontic cleaners was higher than that of tap water (Fig. 2, Table 1). On the other hand, the results of this study demonstrated that the effectiveness of chemical cleaning tablets is limited on a mature biofilm due to the associated structural changes. Compared to a 4-day-old biofilm [16], there was a clear decrease in protein reduction. The results of this study, and the results of previous studies, reinforce the need to combine the use of chemical cleaning tablets with manual brushing to assure adequate biofilm removal. Although the orthodontic cleaning tablets were able to remove some of the biofilm, a significant amount of biofilm remained. Orthodontic cleaners did not promote broad elimination of the biofilm, within the time of use recommended by the manufacture.

The time of immersion is an important factor to consider. He et al. [24] investigated the penetration of chlorhexidine into the inner part of a mature biofilm. They were able to show that a limited penetration time did not result in concentrations necessary to kill the bacteria. Coenye et al. [8] determined the kinetics of killing for *C. albicans* biofilms grown on PMMA specimens. By increasing the time of exposure in a peroxide-based solution (NitrAdine™, Medical Interporous, MSI Laboratories AG, Vaduz, Liechtenstein) a significant reduction of *C. albicans* could be achieved. They stated that the time of exposure to peroxide-based solutions must be long enough for them to penetrate into the deep layers of the biofilm. In the present study, the difference in the cleaning performance between Retainer Brite® vs. Dontodent and Kukis® Xpress, respectively, was significant. While Retainer Brite® with an exposure time of 15 min led to the highest protein reduction, the cleaner Kukis® Xpress with the shortest exposure time of 3 min achieved the lowest protein reduction. However, the difference in protein reduction between Kukis® Xpress and Dontodent was not significant despite a longer exposure time in the cleaning solution for Dontodent. Since only cleaning performance of the use according to the manufacturers’ time instructions was tested, it remains to be clarified whether a longer soaking time in the cleaning solution leads to better cleaning results. Furthermore, it remains to be clarified whether the daily use of orthodontic cleaners can prevent the accumulating formation of biofilm. This question should be investigated in future studies.

## Conclusion

The results of this ex vivo study demonstrate that under the condition of a 7-day-old biofilm, orthodontic cleaners removed some of the biofilm, but a significant amount of biofilm remained. The cleaning performance on a mature biofilm with its associated structural changes is limited. On a mature biofilm the single use of water is ineffective and leads to inadequate cleaning results.
